# Supplementary material for: Complexity of modular neuromuscular control increases and variability decreases during human locomotor development
Source: Commun Biol. 2022 Nov 16;5:1256. doi: 10.1038/s42003-022-04225-8 (PMC9669031; doi:10.1038/s42003-022-04225-8)
Supplement: Supplementary file 2 — Supplementary Material [file 42003_2022_4225_MOESM2_ESM.pdf]

Supplementary information for

Complexity and variability of modular neuromuscular control during human locomotor development

Francesca Sylos-Labini, Valentina La Scaleia, Germana Cappellini, Arthur Dewolf, Adele Fabiano, Irina A. Solopova, Vito Mondi, Yury Ivanenko, Francesco Lacquaniti

This PDF file includes: Supplementary Figures 1 to 7 and Supplementary Table 1

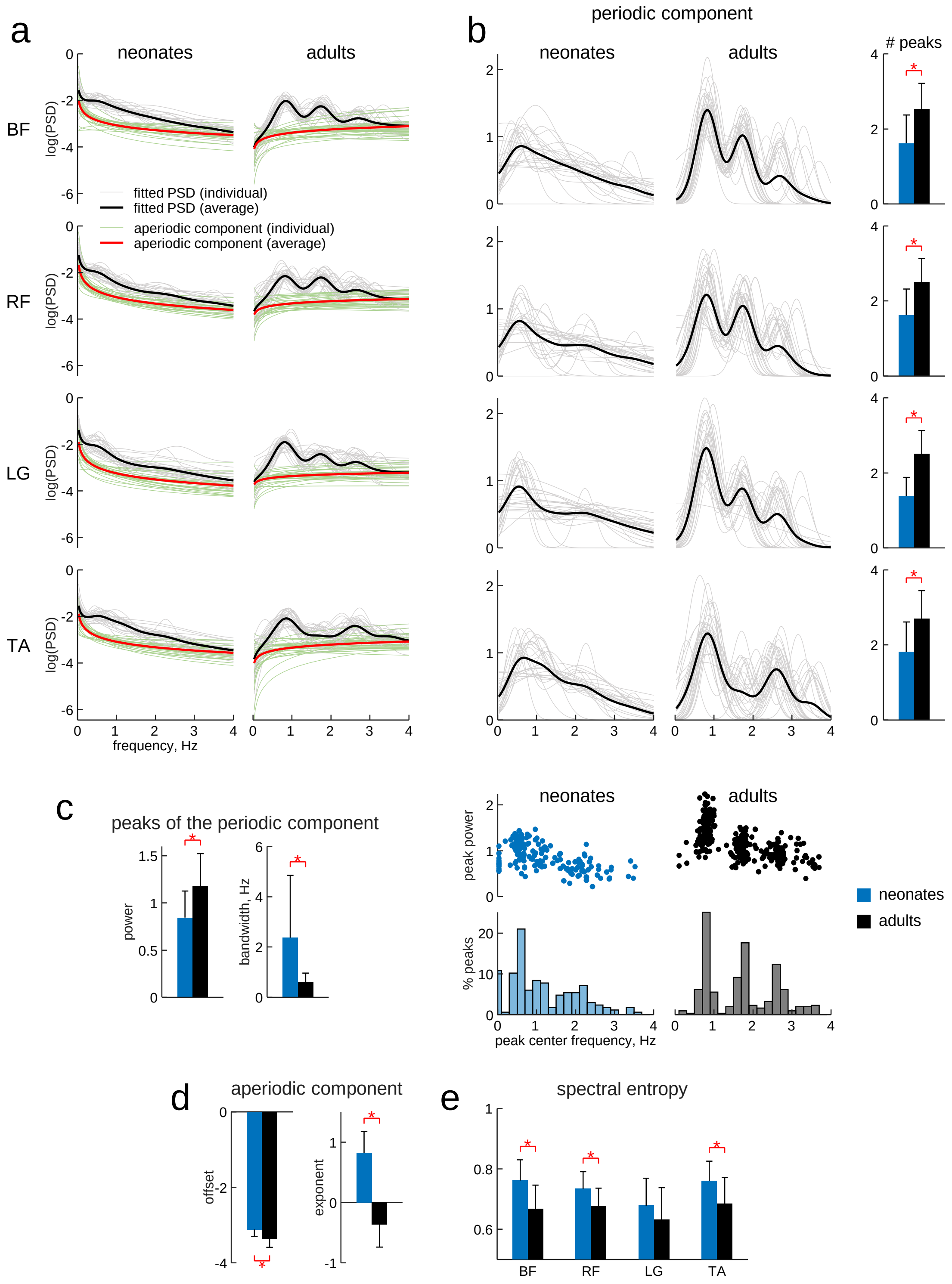

Supplementary Figure 1. Parametrized frequency analysis of EMG data not interpolated over the gait cycle. a Power spectral density (PSD) for all neonates (*left*) and all adults (*right*) was calculated from rectified EMGs for each muscle (*from top to bottom*: BF, RF, LG and TA, right and left leg muscles were pooled together) using Fast Fourier Transform (FFT) and fitted with an algorithm for parameterizing neural PSDs into periodic and aperiodic components[1]. We limited the frequency band of the analyses up to 4 Hz to eliminate boundary effects due to low-pass filtering at 5 Hz. *Grey lines* represent fitted PSDs from individual subjects, *black lines* represent average fitted PSD across subjects, *green lines* represent the aperiodic component from the individual PSDs, *red*

lines represent the average aperiodic component across subjects. In contrast with the time-interpolated EMG data (Fig. 2), in this case the best model for the aperiodic component did not involve the knee parameter (k=0) for both groups. **b** Periodic component of the PSD for all neonates (*left*) and all adults (*centre*), *black lines* represent average across subjects. The average (+SD) number of peaks (defined from the algorithm as the frequency regions of power over and above the aperiodic component) across subjects of each group are illustrated in the *right* column for each muscle. **c** Characteristics of the peaks of the periodic component. The average (+SD) power (*left*) and bandwidth (*right*) of the peaks across subjects and muscles are shown on the *left* side of the panel. The distributions of the peak power (*upper plots*, each point represents a peak, all peaks from all muscles of all subjects are pooled together) and of the percent number of peaks across centre frequencies of the peaks are shown on the *right* side of the panel. Note that, given the high variability of stride duration in neonates, the centre frequency of the main peak is not consistent. **d** Parameters of the aperiodic component. From *left* to *right* average (+SD) offset and exponent of the aperiodic component across subjects and muscles. Notice that both parameters significantly differ between neonates and adults. **e** Average (+SD) spectral entropy across subjects for each muscle in neonates and adults. Asterisks denote significant differences between groups (Wilkoxon rank sum test $p < 0.05$).

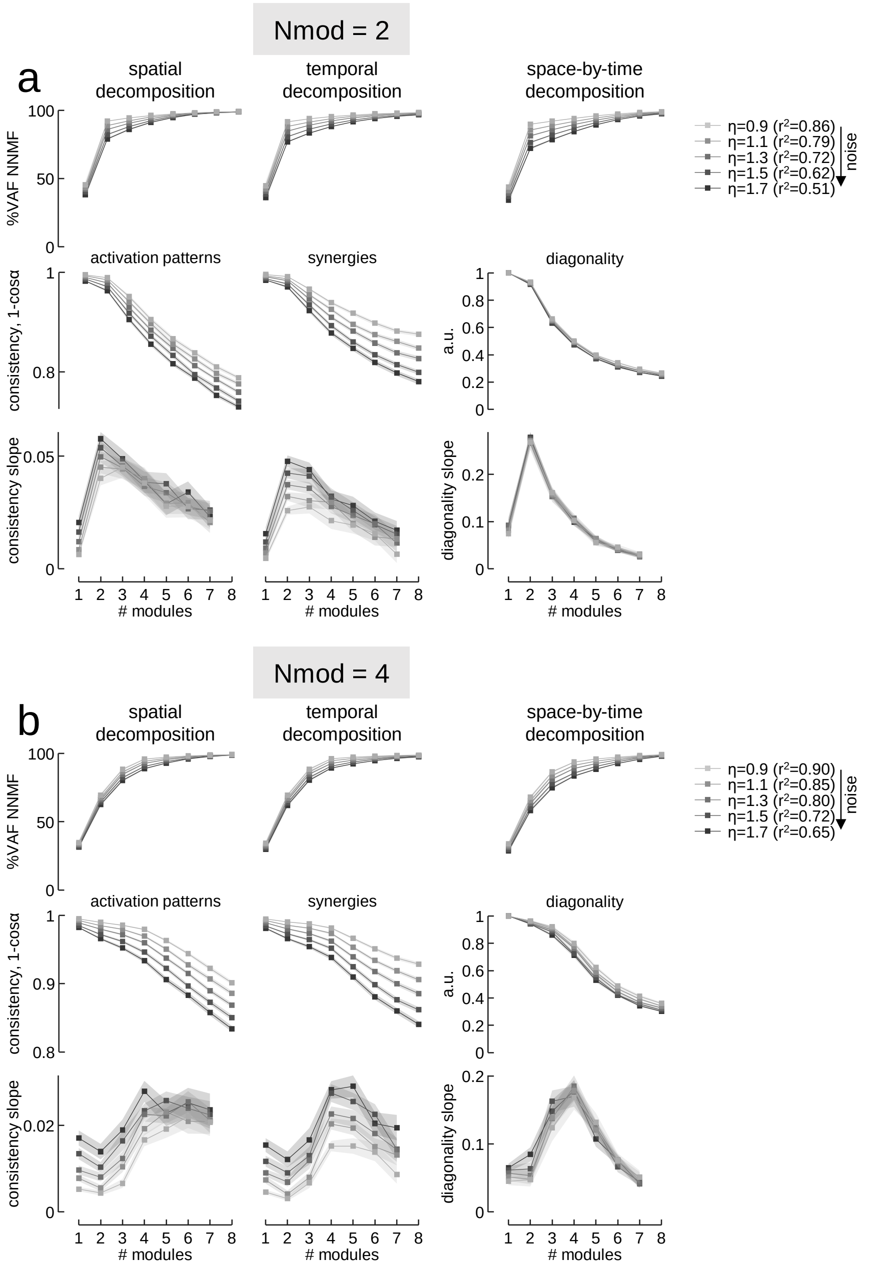

Supplementary Figure 2. Effect of noise on simulated data (same analysis as in Figure 4b but assuming different number of modules: 2 and 4). a Effect of noise on the percent of variance accounted for (VAF, *upper*) by the reconstruction of simulated EMGs using three different decomposition methods (*from left to right*, spatial, temporal and space-by-time decomposition) and on the similarity measures (*middle*) and their slope (*lower*): average similarity of the activation patterns across strides for spatial decomposition, average similarity of synergies across strides for temporal decomposition and average diagonality of the activation coefficients matrix across strides for space-by-time decomposition (*from left to right*). The simulated EMGs were constructed, by an adaptation of the method proposed by Tresch et al.[2], using the corresponding equation for each model to calculate noiseless data value $m^s(t)$ and adding signal-dependent noise with $SD = \eta \cdot m^s(t)$, where η is the slope of the relationship between the SD and noiseless data value. We constructed sets of 8 EMGs for 7 strides starting from 2 modules (Nmod) composed of constant synergies and basic patterns as in Figure 4a. Each simulation was performed using increasing values of η for 100 times (averages across simulations and confidence intervals – as shaded areas – are shown in the graphs). **b** Same format as panel a with simulated data constructed starting from 4 modules. Notice that VAF

was significantly affected by noise, making it difficult to distinguish the exact number of modules (Nmod=2 for panel a and Nmod=4 for panel b) used to generate the simulated EMGs for higher levels of noise for each decomposition method, while the similarity measures showed a substantial reduction going from 2 to 3 modules in panel a and from 4 to 5 modules in panel b regardless of the level of noise.

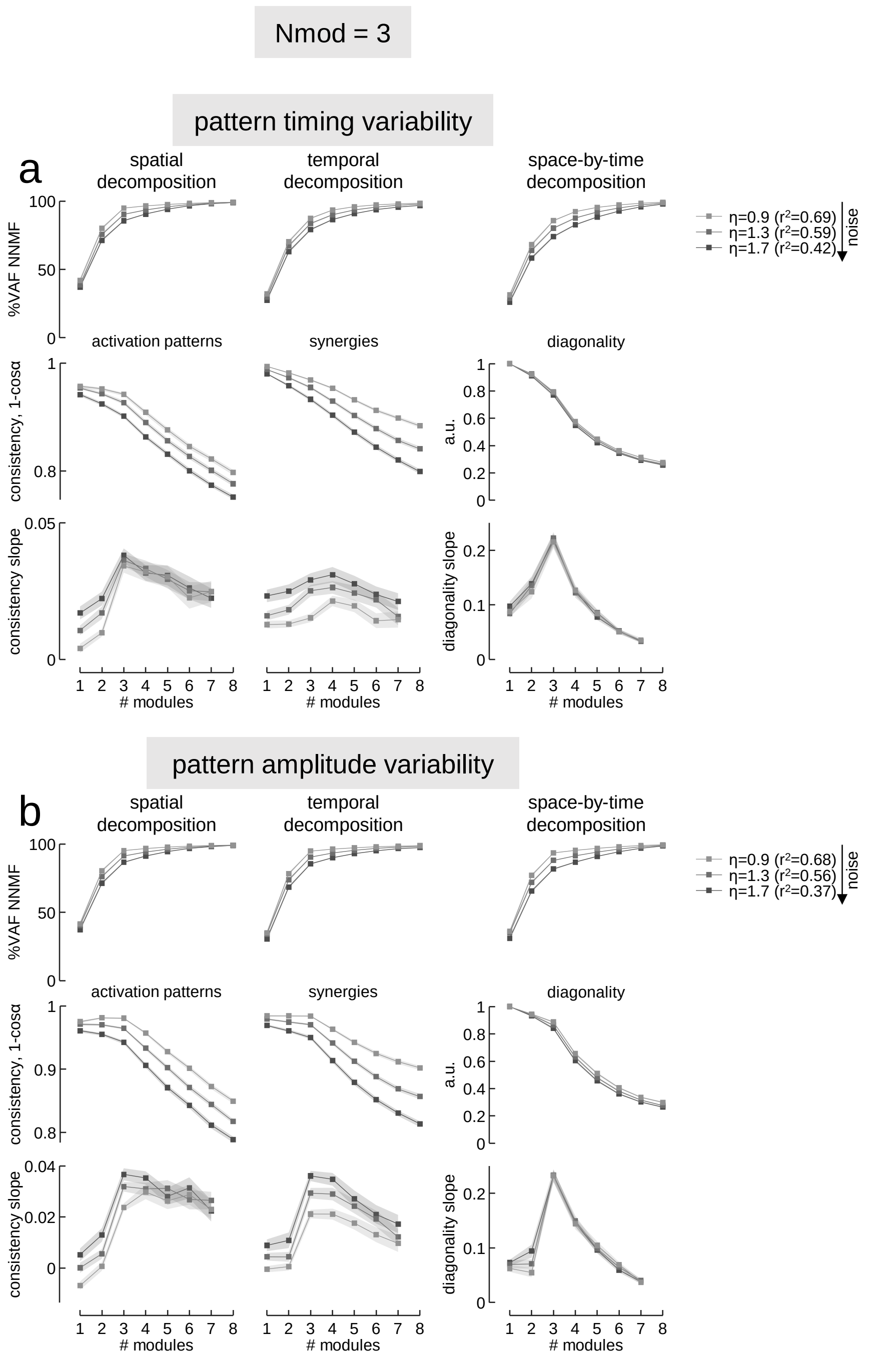

Supplementary Figure 3. Effect of cycle-to-cycle variability on simulated data (same analysis as in Figure 4b but adding timing and amplitude variability to the basic activation pattern). a Effect of noise on the percent of variance accounted for (VAF, *upper*) by the reconstruction of simulated EMGs using three different decomposition methods (*from left to right*, spatial, temporal and space-by-time decomposition) and on the similarity measures (*middle*) and their slope (*lower*): average similarity of the activation patterns across strides for spatial decomposition, average similarity of synergies across strides for temporal decomposition and average diagonality of the activation coefficients matrix across strides for space-by-time decomposition (*from left to right*). The simulated EMGs were constructed, by an adaptation of the method proposed by Tresch et al.[2], using the corresponding equation for each model to calculate noiseless data value $m^s(t)$ and adding signal-

dependent noise with $SD = \eta \cdot m^s(t)$, where η is the slope of the relationship between the SD and noiseless data value. We constructed sets of 8 EMGs for 7 strides starting from 3 modules (Nmod) composed of constant synergies and basic patterns independently shifted across the gait cycle by a random interval drawn from a normal distribution of mean 0% and standard deviation 5% of the gait cycle. Each simulation was performed using increasing values of η for 100 times (averages across simulations and confidence intervals – as shaded areas – are shown in the graphs). **b** Same format as panel a with simulated data whose basic patterns were independently scaled in amplitude by a random factor drawn from a normal distribution of mean 1 and standard deviation 0.3 of their amplitude. Note that VAF was significantly affected by cycle-to-cycle variability, making it difficult to distinguish the exact number of modules (Nmod=3) used to generate the simulated EMGs for higher levels of noise for each decomposition method, while the similarity measures showed a substantial reduction going from 3 to 4 modules regardless of the level of noise.

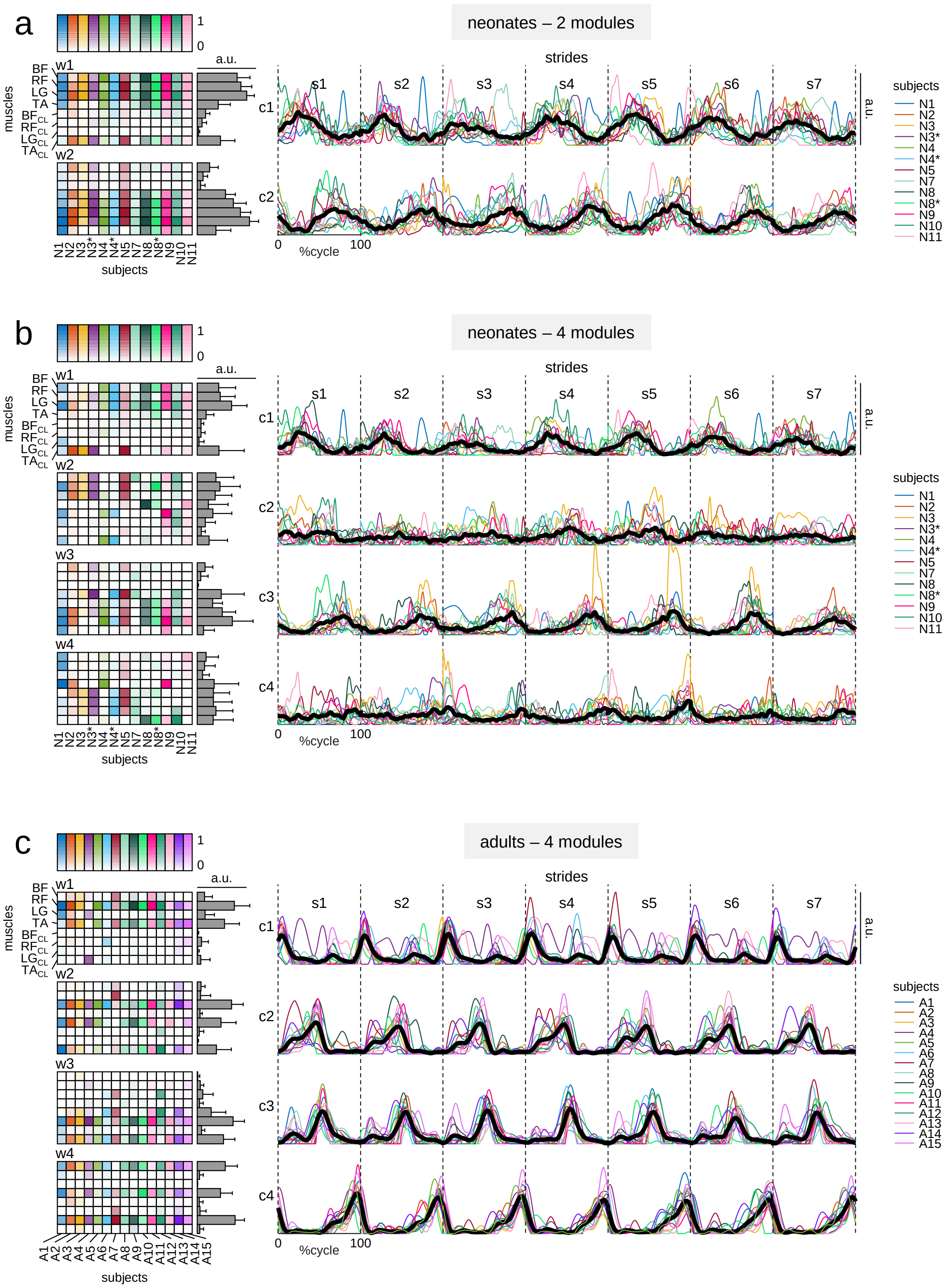

Supplementary Figure 4. Spatial decomposition. a Result of the spatial decomposition of EMG data of neonates using two modules. *Left column*: muscle synergies (w_1 and w_2, *from top to bottom*), each column represents a single neonate, and each row represents a muscle (BF: Biceps Femoris, RF: Rectus Femoris, LG: Lateral Gastrocnemius, TA: Tibialis Anterior, CL: Contralateral Leg), the intensity of the colour is proportional to the muscle weight, the average (+SD) values are illustrated

through grey bars on the right. *Right column*: basic activation patterns (c_1 and c_2, *from top to bottom*) across 7 strides (s_1-s_7), each colour represents a single neonate, black lines indicate the average across subjects. The modules of each subject are grouped in order to attain the minimum distance (1-scalar product) between the average (across strides) of the basic activation patterns of all subjects. **b** Spatial decomposition of EMG data of neonates using four modules, same format as panel a. **c** Spatial decomposition of EMG data of adults using four modules, same format as panels a and b. Note that, despite four modules in neonates account on average for 83% ($CI_{95\%}$ 81%÷85%) of the variance of the EMG data [in neonates two modules account on average for 62% ($CI_{95\%}$ 59%÷66%) of the variance, and in adults four modules account on average for 83% ($CI_{95\%}$ 81%÷84%) of the variance], these modules are visibly less consistent across strides and across subjects (the average across subjects of both synergies and basic activation patterns are flatter) compared with the decomposition with two modules in neonates (panel a) and four modules in adults (panel c).

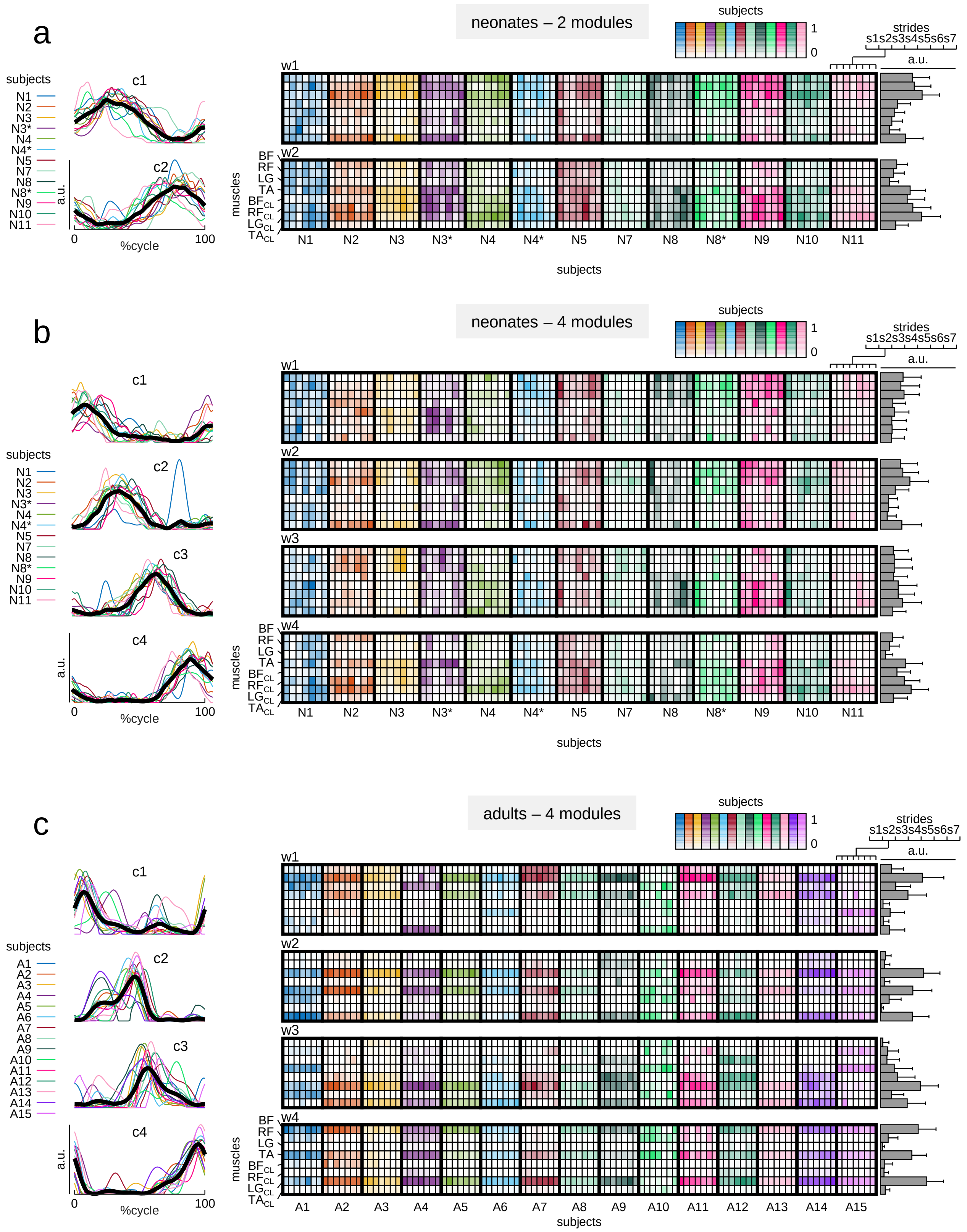

Supplementary Figure 5. Temporal decomposition. a Result of the temporal decomposition of EMG data of neonates using two modules. *Left column*: basic activation patterns (c_1 and c_2, *from top to bottom*), each coloured line represents a single neonate, black lines indicate the average across subjects. *Right column*: muscle synergies (w_1 and w_2, *from top to bottom*), each column represents a single stride (s_1-s_7) of each neonate (in different colours), and each row represents a muscle (BF: Biceps Femoris, RF: Rectus Femoris, LG: Lateral Gastrocnemius, TA: Tibialis Anterior, CL:

Contralateral Leg), the intensity of the colour is proportional to the muscle weight, the average (+SD) values are illustrated through grey bars on the right. The modules of each subject are grouped in order to attain the minimum distance (1-scalar product) between the basic activation patterns of all subjects. **b** Temporal decomposition of EMG data of neonates using four modules, same format as panel a. **c** Temporal decomposition of EMG data of adults using four modules, same format as panels a and b. Note that, despite four modules in neonates account on average for 72% ($CI_{95\%}$ 69%÷74%) of the variance of the EMG data [in neonates two modules account on average for 44% ($CI_{95\%}$ 41%÷47%) of the variance, and in adults four modules account on average for 81% ($CI_{95\%}$ 77%÷83%) of the variance], these modules are visibly less consistent across strides and across subjects (the average synergies across subjects are flatter) compared with the decomposition with two modules in neonates (panel a) and four modules in adults (panel c).

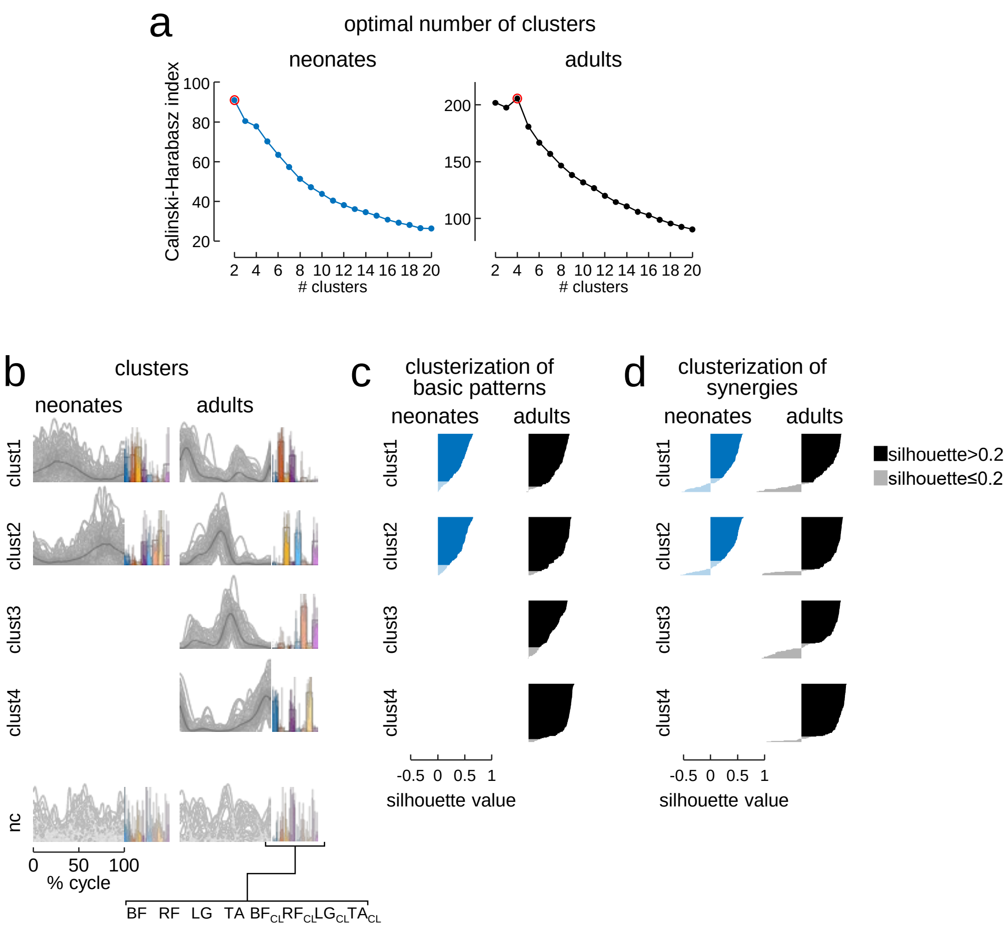

Supplementary Figure 6. Cluster analysis. a Evaluation of the optimal number of clusters using the Calinski-Harabasz method in neonates (*blue* plot on the *left*) and adults (*black* plot on the *right*). **b** Clusters of activation patterns (S > 0.2) from single cycles of all subjects of each group in *grey*, average patterns in *black*. Corresponding synergies weights (S > 0.2) for single cycles in *colour*, average values as *empty bars*. Patterns and synergies are plotted only if S > 0.2 in >15% of cases. (*Lower*) Not-clustered (nc, S ≤ 0.2) activation patterns (*light gray*) and associated weights. **c** Silhouettes of activation patterns ranked in decreasing order for the single strides of panel b (below-threshold silhouettes in *light colour*). **d** Silhouettes of synergies weights in the same format as panel c.

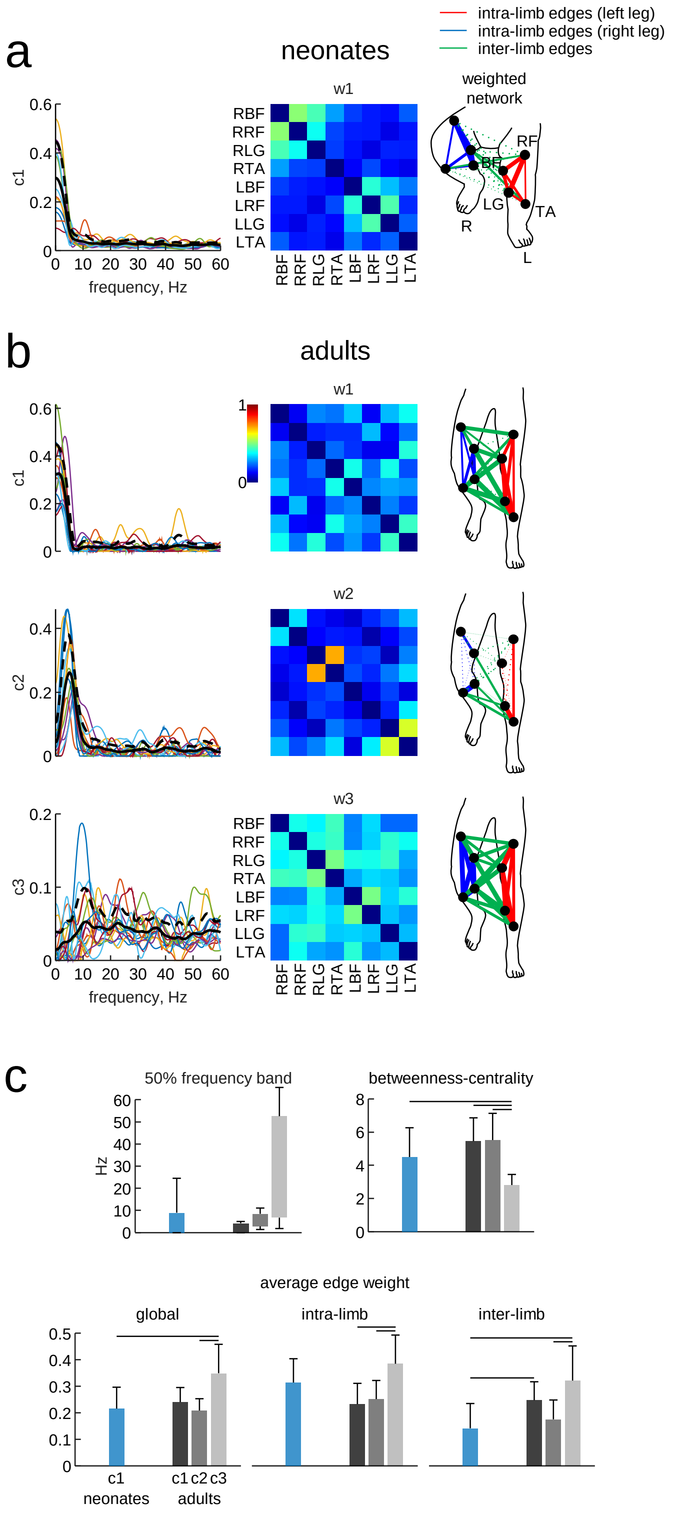

Supplementary Figure 7. Inter-muscular coherence networks for neonates and adults (same analysis as in Figures 8-9 but assuming a different number of components, 1 or 3). a First component (accounting on average 64% of VAF, 95% confidence interval 49%÷75%) obtained with non-negative matrix factorization of the frequency content of the coherence spectra of all muscle combinations in neonates (*black lines* denote average across subjects +SD in *dashed black lines*). The *central column* shows the average loadings (w_1) of the corresponding frequency component across subjects. These loadings give the strength of the edges between the 8 nodes of each muscle weighted network (n_1, *right column*), connection strength is reflected by the width of the lines. **b** First three components (accounting on average 78% of VAF, 95% confidence interval 72%÷82%) obtained with

non-negative matrix factorization of the frequency content of the coherence spectra of all muscle combinations in adults (same format as panel a). The components are ordered (*from top to bottom*) based on the peak of the frequency components (c_1-c_3, *left* column). **c** Summary statistics of inter-muscular coherence networks. Network metrics were used to statistically compare the muscle networks across groups and frequencies: average (±SD) 50% frequency band across subjects (*upper row*, *left panel*), average (+SD) betweenness-centrality across subjects (*upper row*, *right panel*) and average (+SD) edge weight across subjects (*lower row*) considering all network edges (global, *left panel*), only the intra-limb edges (intra-limb, *central panel*) or only the inter-limb edges (inter-limb, *right panel*). Horizontal red lines denote significant differences between networks (post-hoc Tukey-Kramer multiple comparison $p < 0.05$).

Supplementary Table 1. Walking speeds recorded in individual subjects. Age is expressed in days for the neonates, in months for the infants and toddlers and in years for the preschoolers and adults, weight in kilograms, limb length in meters, and speed in meters per seconds (grd. overground walking).

Neonates

id	sex	age	weight	limb length	speed											
					grd.	0.03	0.05	0.10	0.15	0.20	0.25	0.30	0.40	0.50	0.60	1.1
N1	f	1	3.5	0.19			x									
N2	f	1	3.4	0.19			x									
N3	f	2	3.1	0.17			x	x								
N4	f	2	2.9	0.18			x	x								
N5	m	2	3.3	0.20		x										
N6	f	6	2.9	0.18			x									
N7	m	9	3.0	0.19			x									
N8	m	11	2.7	0.18			x	x								
N9	f	11	3.2	0.20			x									
N10	f	11	3.4	0.19		x										
N11	m	14	3.6	0.22			x									

Infants (g1-g4)

id	sex	age	weight	limb length	speed											
					grd.	0.03	0.05	0.10	0.15	0.20	0.25	0.30	0.40	0.50	0.60	1.1
I1	f	4	5.9	0.21	x											
I2	f	4.1	6.1	0.22			x	x								
I3	m	4.2	6.5	0.22			x	x								
I4	m	5	6.8	0.23	x											
I5	f	5	6.5	0.23	x											
I6	f	5.4	5.5	0.21			x									
I7	m	5.7	6.5	0.24				x								
I8	f	6.7	7.7	0.24					x	x	x					
I9	m	7	8.3	0.25	x											
I10	m	7.5	9.0	0.25	x											
I11	m	7.5	7.1	0.23	x											
I12	m	7.5	9.3	0.26	x											
I13	m	7.5	8.5	0.25	x											
I14	f	7.6	8.4	0.25				x	x	x		x	x			
I15	f	9.5	7.5	0.23	x											
I16	m	9.6	9.9	0.27				x	x	x		x	x	x		
I17	m	9.7	11.2	0.28				x	x			x	x	x	x	
I18	m	9.8	10.0	0.27					x	x			x	x		
I19	m	9.9	8.4	0.25						x		x	x	x		
I20	m	10.2	10.0	0.28				x	x	x		x	x			
I21	m	10.4	10.6	0.28			x		x	x			x	x	x	
I22	m	10.5	12.0	0.27	x											
I23	m	10.5	12.5	0.27	x											
I24	m	10.9	9.0	0.28					x	x		x	x	x	x	
I25	m	11.9	12.0	0.28				x	x	x		x	x	x		
I26	m	12.4	8.8	0.25								x	x	x		
I27	f	12.5	11.1	0.28						x						
I28	m	12.9	7.7	0.29				x	x	x	x	x	x		x	
I29	f	13.6	13.0	0.28					x	x		x	x	x	x	
I30	f	13.7	10.4	0.27				x	x	x		x	x	x	x	

Toddlers

id	sex	age	weight	limb length	grd.	speed											
						0.03	0.05	0.10	0.15	0.20	0.25	0.30	0.40	0.50	0.60	1.1
I31	m	11	10.0	0.26	x											
I32	f	12	8.5	0.25	x											
I33	f	12	11.0	0.26	x											
I34	f	12	9.2	0.26	x											
I35	f	12	8.5	0.25	x											
I36	m	12.3	9.2	0.26						x						
I37	f	12.6	10.0	0.29									x	x	x	
I38	m	12.8	9.6	0.29				x	x	x		x	x	x	x	
I39	f	13	10.5	0.27	x											
I40	f	13.2	9.6	0.30	x											
I41	m	13.6	10.0	0.27				x	x	x	x	x	x	x		
I42	m	14.4	9.6	0.28						x						
I43	f	14.4	9.2	0.29	x											
I44	m	14.4	9.1	0.26	x											
I45	m	14.4	9.1	0.28	x											

Preschoolers

id	sex	age	weight	limb length	grd.	speed											
						0.03	0.05	0.10	0.15	0.20	0.25	0.30	0.40	0.50	0.60	1.1
I46	f	2.1	6.3	0.36	x											
I47	f	2.6	15.0	0.38	x											
I48	m	2.8	12.5	0.36	x											
I49	m	2.9	12.4	0.35	x											
I50	f	3	12.8	0.34	x											
I51	m	3.3	15.0	0.39	x											
I52	m	4	17.4	0.44	x											
I53	m	4.2	14.4	0.44	x											

Adults

id	sex	age	weight	limb length	grd.	speed											
						0.03	0.05	0.10	0.15	0.20	0.25	0.30	0.40	0.50	0.60	1.1
A1	f	19	62	0.74												x
A2	f	20	67	0.74												x
A3	f	20	56	0.65												x
A4	m	21	75	0.79												x
A5	m	22	62	0.75												x
A6	f	24	62	0.73												x
A7	m	24	90	0.85												x
A8	m	24	73	0.82												x
A9	m	25	72	0.81												x
A10	f	25	55	0.64												x
A11	f	26	68	0.72												x
A12	m	28	70	0.89												x
A13	m	35	65	0.75												x
A14	f	44	65	0.78												x
A15	m	50	85	0.85												x

Supplementary References

1. Donoghue, T. *et al.* Parameterizing neural power spectra into periodic and aperiodic components. *Nat. Neurosci.* **23**, 1655–1665 (2020).
2. Tresch, M. C., Cheung, V. C. K. & d'Avella, A. Matrix factorization algorithms for the identification of muscle synergies: evaluation on simulated and experimental data sets. *J. Neurophysiol.* **95**, 2199–2212 (2006).
