# Supplementary material for: Complexity of modular neuromuscular control increases and variability decreases during human locomotor development
Source: Commun Biol. 2022 Nov 16;5:1256. doi: 10.1038/s42003-022-04225-8 (PMC9669031; doi:10.1038/s42003-022-04225-8)
Supplement: Supplementary file 5 — Reporting Summary [file 42003_2022_4225_MOESM5_ESM.pdf]

nature portfolio

Corresponding author(s): Francesca Sylos-Labini

Last updated by author(s): Oct 18, 2022

Reporting Summary

Nature Portfolio wishes to improve the reproducibility of the work that we publish. This form provides structure for consistency and transparency in reporting. For further information on Nature Portfolio policies, see our Editorial Policies and the Editorial Policy Checklist.

Statistics

For all statistical analyses, confirm that the following items are present in the figure legend, table legend, main text, or Methods section.

n/a	Confirmed	
☐	☒	The exact sample size (*n*) for each experimental group/condition, given as a discrete number and unit of measurement
☐	☒	A statement on whether measurements were taken from distinct samples or whether the same sample was measured repeatedly
☐	☒	The statistical test(s) used AND whether they are one- or two-sided<br>*Only common tests should be described solely by name; describe more complex techniques in the Methods section.*
☐	☒	A description of all covariates tested
☐	☒	A description of any assumptions or corrections, such as tests of normality and adjustment for multiple comparisons
☐	☒	A full description of the statistical parameters including central tendency (e.g. means) or other basic estimates (e.g. regression coefficient) AND variation (e.g. standard deviation) or associated estimates of uncertainty (e.g. confidence intervals)
☐	☒	For null hypothesis testing, the test statistic (e.g. *F*, *t*, *r*) with confidence intervals, effect sizes, degrees of freedom and *P* value noted<br>*Give P values as exact values whenever suitable.*
☒	☐	For Bayesian analysis, information on the choice of priors and Markov chain Monte Carlo settings
☒	☐	For hierarchical and complex designs, identification of the appropriate level for tests and full reporting of outcomes
☐	☒	Estimates of effect sizes (e.g. Cohen's *d*, Pearson's *r*), indicating how they were calculated

Our web collection on statistics for biologists contains articles on many of the points above.

Software and code

Policy information about availability of computer code

Data collection	We used a 3D SIMI motion capture system (Munich, Germany, software Simi Motion 8.5.3) to record stepping on treadmill in neonates, and a 3D Vicon Bonita system (Oxford, UK, software Nexus 12.1) in infants who stepped on treadmill and adults.<br>In neonates, surface EMG activities were recorded using the wireless Zerowire system (Aurion Srl, Italy, software Wave 1.1). In infants and adults, EMGs were recorded by means of the Trigno Wireless EMG System (Delsys Inc., Boston, MA) using the Vicon Nexus 12.1 software.
Data analysis	All data analysis was performed using custom-written programs in Matlab R2018a (MathWorks, Natick, MA).

For manuscripts utilizing custom algorithms or software that are central to the research but not yet described in published literature, software must be made available to editors and reviewers. We strongly encourage code deposition in a community repository (e.g. GitHub). See the Nature Portfolio guidelines for submitting code & software for further information.

Data

Policy information about availability of data

All manuscripts must include a data availability statement. This statement should provide the following information, where applicable:

- Accession codes, unique identifiers, or web links for publicly available datasets
- A description of any restrictions on data availability
- For clinical datasets or third party data, please ensure that the statement adheres to our policy

All data generated or analysed during this study are included in this published article (and its supplementary information files). Additional data are available from the corresponding author upon reasonable request.

Human research participants

Policy information about studies involving human research participants and Sex and Gender in Research.

Reporting on sex and gender	Sex of all participants is reported in the Tables.
Population characteristics	Table 1 gives the average characteristics of the eight different groups: full-term neonates (Apgar score ≥8 at 1 and 5 min, uneventful delivery and perinatal history), infants of 6 different age groups, group 1 (g1, age range 4-6 months), group 2 (g2, 6-8 months), group 3 (g3, 8-10 months), group 4 (g4, 10-14 months), toddlers (12-15 months), pre-schoolers (24-48 months), and adults. Supplementary Table 1 gives detailed characteristics for all individuals separately.
Recruitment	Neonates were recruited and studied in the hospital well-baby maternity ward. Infants and adulths were recruited at the Santa Lucia Foundation and a subset of infants at the Veltischev Research and Clinical Institute for Paediatrics of the Pirogov Russian National Research Medical University. A parent for the child and all adult participants provided informed written consent to participate in the study after the nature and possible consequences of the study were explained. All procedures were not invasive and did not involve any specific risk. All experiments with children were performed with the direct participation or supervision of an MD.
Ethics oversight	The Research Ethics Committee of Azienda Sanitaria Locale (Local Health Centre) Roma C approved the experiments with the neonates (protocol CEI/15843 study n. 609, and protocol 27593, study n. 38.15). The Research Ethics Committee of Santa Lucia Foundation approved the experiments with a subset of infants and all adults (protocol CE/AG4/PROG.341-01). The Research Ethics Committee of the Veltischev Research and Clinical Institute for Paediatrics of the Pirogov Russian National Research Medical University approved the experiments for another subset of infants (protocol n. 14/18).

Note that full information on the approval of the study protocol must also be provided in the manuscript.

Field-specific reporting

Please select the one below that is the best fit for your research. If you are not sure, read the appropriate sections before making your selection.

☒ Life sciences ☐ Behavioural & social sciences ☐ Ecological, evolutionary & environmental sciences

For a reference copy of the document with all sections, see nature.com/documents/nr-reporting-summary-flat.pdf

Life sciences study design

All studies must disclose on these points even when the disclosure is negative.

Sample size	The participants were selected from a larger sample because they had full EMG recordings from all 8 tested muscles of at least 7 strides. However, the results obtained using the cluster analysis to identify similar muscle synergies and activation patterns across all recorded strides of all participants of each age group on the data from this subsample replicated the results obtained from the entire sample published in our previous paper (Sylos-Labini et al., PNAS, 2020).
Data exclusions	Children who did not step were excluded from the study, since stepping was a pre-requisite.
Replication	In order to verify the replicability of the results we simulated several datasets with varying amount of noise, matching real data but with known dimensionality). We generated 100 datasets on which we applied the decomposition algorithms (varying the number of modules from 1 to 8), and calculated VAF and consistency measures. The same analysis was performed on 100 structureless datasets obtained by randomly shuffling all samples of the simulated EMGs, independently for each channel.
Randomization	The allocation of participants into experimental groups was not random since it was based on the subject's age.
Blinding	The blinding of group allocation during data collection was not possible since the age of the participants was evident to the experimenters. However, all analyses were performed in an automatic mode, so that the results were effectively blinded.

Reporting for specific materials, systems and methods

We require information from authors about some types of materials, experimental systems and methods used in many studies. Here, indicate whether each material, system or method listed is relevant to your study. If you are not sure if a list item applies to your research, read the appropriate section before selecting a response.

Materials & experimental systems

n/a	Involved in the study
☒	☐ Antibodies
☒	☐ Eukaryotic cell lines
☒	☐ Palaeontology and archaeology
☒	☐ Animals and other organisms
☒	☐ Clinical data
☒	☐ Dual use research of concern

Methods

n/a	Involved in the study
☒	☐ ChIP-seq
☒	☐ Flow cytometry
☒	☐ MRI-based neuroimaging
